# Academic librarian support for patient-centred and inclusive medical education curricula: a case report

**DOI:** 10.5195/jmla.2025.2106

**Published:** 2025-10-23

**Authors:** Jackie Phinney, Leanne Picketts, Lynette Reid

**Affiliations:** 1 j.phinney@dal.ca, Dalhousie University Libraries/Dalhousie Medicine New Brunswick, Saint John, N.B., Canada; 2 leanne.picketts@dal.ca, Centre for Collaborative Clinical Learning and Research, Undergraduate Medical Education, Dalhousie University, Halifax, N.S.; 3 lynette.reid@dal.ca, Department of Bioethics, Dalhousie University, Halifax N.S.

**Keywords:** Medical education, Academic librarianship, Case-Based Learning, EDIA, Patient-Centred Care

## Abstract

**Background:**

Medical educators are increasingly aware of the need for patient-centred and inclusive curricula. Collaboration paired with sound evidence can facilitate efforts in this area. Librarians are well-equipped to help move this work forward, as their skills and expertise can support educators through the process of revising learning materials that will incorporate timely and socially accountable information.

**Case Presentation:**

This case report describes an initiative at one Canadian medical school, whereby a health sciences librarian joined an interdisciplinary working group to support the updating of case-based learning materials for the undergraduate medical curriculum. These materials were revised with an anti-oppressive and patient-centred lens, and as an embedded member of the working group the librarian provided on-demand literature searches, participated in conversations regarding the importance of critical appraisal skills, and consulted on sustainable access to electronic materials used in the cases. From this experience and close collaboration, lessons which enhanced their practice and stronger relationships emerged for the librarian.

**Conclusions:**

Involving librarians' expertise in updating learning materials provides many benefits to curriculum developers and presents opportunities for liaison librarians to engage with their faculties more closely. Promoting patient-centredness and inclusivity is an ongoing process, and academic health sciences librarians can apply their expertise to curricular initiatives such as the one described here, while librarians working in clinical settings can support these efforts through specialized forms of teaching and outreach.

## BACKGROUND

For many years, physicians have been called upon to assume the role of health advocate for those communities negatively affected by the social/structural determinants of health [1], and both the Association of American Medical Colleges (AAMC) and the Association of Faculties of Medicine of Canada (AFMC) have demonstrated their commitment to this call [2,3]. In response to this, medical schools have worked to address curricular gaps related to equity, diversity, inclusion, and accessibility (EDIA) [4,5], but much work is still needed to seamlessly integrate these topics throughout the medical school curricula in a meaningful way [6].

Case-based learning (CBL) has been a prominent feature of the undergraduate medical curriculum at Dalhousie University for several years, and its application is found primarily within the first two years (pre-clerkship) of a four-year M.D. program. During multiple weekly, small-group CBL sessions, students discuss and debate patient scenarios under the guidance of faculty tutors who offer help using a guide (prepared by the case authors) that contains detailed prompts and additional information to share throughout the discussion. While cases are regularly reviewed for clinical currency, periodic deeper revisions of the curriculum's pedagogy in relation to social accountability are undertaken in accordance with accreditation cycles. In 2021-22, working groups (including working groups on Priority Communities, Anti-Oppressive Practice, Planetary Health, Addictions Medicine, and Generalism) reviewed the entire curriculum and put forward several recommendations for curricular change. These recommendations led to the subsequent creation of a new working group that was tasked with updating CBL materials. From this experience, new partnerships were created while existing ones, such as that between the university's health sciences library and the Faculty of Medicine, were strengthened.

Librarians are demonstrated partners in curriculum reform as well as social justice efforts. The literature describes academic librarians collaborating with faculty to instruct on critical information literacy [7], as well as making efforts to represent marginalized groups in their collections [8]. Within the academic health sciences library sector, there are examples of librarians working alongside faculty to expand curriculum content [9] and participating in team-based collaboration to revise cases depicting patients [10]. Quite recently, efforts to diversify teaching images in undergraduate medical curricula have involved collaboration between students, faculty, and librarians [11], thereby demonstrating the timeliness of this work and librarians' ability to collaborate with different stakeholder groups in support of systemic change.

This case report describes how a health sciences librarian integrated within a medical school working group to support efforts to update CBL materials for greater inclusivity and patient-centredness. It incorporates the perspectives of the librarian (JP), the working group Chair (LR), and Equity, Diversity, Inclusion and Accessibility (EDIA) Curriculum Reviewer (LP) (also referred to as the Reviewer) to demonstrate the value of including the library in the update and development of learning materials. The work described in this case report aligns with active calls for health sciences librarians to interrogate the profession's historical role in perpetuating harm against marginalized groups [12] and encourages health educators to consider how their library can supplement EDIA initiatives locally.

## CASE PRESENTATION

As previously noted, working groups were tasked with putting forth recommendations for revisions to the medical curriculum. A key recommendation adopted with respect to CBL was to diversify the patients represented in CBL cases and revise the cases and related curriculum materials (including tutor notes) to remove stigmatizing language. To address these recommendations, the Case Diversification Working Group was established in 2022. It was comprised of twenty-five members including clinicians/physicians, faculty members from the humanities and the biomedical and social sciences, with medical and other students across disciplines, and staff involved in community-engaged service learning. The group was tasked with reviewing and updating all CBL materials (approximately 200 cases) across the pre-clerkship years of the four-year medical curriculum. Case re-writes were largely completed by the previously noted Reviewer (LP) and the working group's Chair (LR) with regular input drawing on the expertise of the working group. The Chair and Reviewer also consulted external clinical and research experts in specific areas and collaborated with the program's Community Engaged Service Learning Program to carry out community engagement. Using an established framework to guide outreach efforts [13], engagement included (for example) outreach with New Brunswick immigration service groups, consultation with the African Nova Scotian Sisterhood and Brotherhood through the faculty's new Black Health Academic Director, and case collaboration with a newly formed provincial intersex society. Noteworthy goals for case revisions included challenging race-based medicine [14], integrating sex and gender diversity [15], portraying clinical care for patients with disabilities [16], and enriching the representation of patient-centred care [17]. In planning to conduct this work, core language used to frame this project's goals was thoroughly considered to ensure contextual understanding that guided the group's processes (see Table 1).

**Table 1:** Key terms that framed case diversification efforts, along with their working definition and how they were operationalized.

Term	Working Definition	How we applied it (examples)
Anti-oppressive practice	Anti-oppressive practice has been used as a framework for pedagogy and for health care provision (or both). Health care education for anti-oppressive practice develops an understanding of the *systemic* nature of *multiple* forms of oppression [19] such as colonialism, racism, sexism, cisheteronormativism, ableism, etc., particularly insofar as these forms of oppression intersect and constitute social determinants of health. It includes specific approaches to providing trauma-informed, inclusive, and affirming care. Anti-oppressive pedagogy involves critical analysis, self-reflection, and de-centering hierarchical teaching relations.	Made all patients' social identities explicit, and diversified these identities. Diversified and gave short descriptions of caregiver and health care provider social identities. Removed/replaced biased and stigmatizing language and frameworks. Integrated discussion questions inviting critical reflection on structural determinants of health and medical complicity in structures of oppression (e.g. how medical science supported racism or enforced sex/gender binaries). Modelled and taught trauma-informed care and advocacy. Removed reference to people coming from the “third world” or “developing countries,” or of the “industrialized” world and replaced where relevant with “low income” or “middle income” countries.
Anti-racism	Anti-racism is “the active process of identifying and eliminating racism by critically evaluating and reforming systems, institutional structures, policies, and language, with the goal of redistributing power equitably” [20 p2]. Anti-racist medical education specifically includes critical reflection on the history of medical racism, understanding race as a social construct and racism as a social determinant of health, addressing the biasing effects of common labelling practices in medical education and case presentation, recognizing racism as attitudinal, interpersonal, institutional, and structural, and developing strategies for challenging all forms of racism.	Identified instances of race-based medicine, and provided updated evidence reviews to inform discussions with case authors and curriculum leaders towards replacing “race-based“ with “race-conscious” medicine. Ensured representation of diverse skin of colour in images and raised awareness of the biasing effects of identifying diagnoses with individuals (e.g. “Lou Gherig's Disease” and the underdiagnosis of ALS in Black persons). Revised descriptions of skin tone for diagnosis (e.g. descriptions of cyanosis, jaundice, striation) that are inaccurate outside of white individuals. Directly challenged common false beliefs about the relative prevalence of substance use disorder by racialized identity. Identified and revised instances where the social determinants of health were presented as individual risk factors and revised to present racism as a structural determinant of health and model advocacy to address its effects.
Decolonization	Decolonization is a social process, specific to a given context, of dismantling settler dominance in sovereignty, land, and knowledge systems. Decoloniality in pedagogy emphasizes reflexivity, dismantling hierarchical teaching, and centering oppressed knowledge systems [19]. Our Canadian context is that of a settler state on Indigenous lands, where Indigenous resurgence [21] meets state efforts towards “reconciliation” [22] “Decolonization is not a metaphor” [23 p1]: our cases *reflect* the broader societal context. Restructuring colonial power relations in the institution and classroom, key aspects of decolonializing pedagogy [19], were beyond the scope of this project.	Indigenous cases written by the program's new Indigenous Health Academic Lead. Portrayed culture and the structural determinants of health in the transformational context of Indigenous resurgence. Introduced learners to the growing Indigenous governance of local health and social care systems. Portrayed the harms of ongoing settler colonialism. Reinforced the concept of “two-eyed seeing” [24 p70]. Modelled trauma-informed care. Educated for strategies to resist specific expressions of anti-Indigenous racism.
Patient-centered	Patient-centred cases portray patients in the context of their families and communities, as intersectional and subject to multiple structural determinants of health. Their experience of health and illness is longitudinal and personal. Patients receive education and counselling (as well as diagnosis and treatment) from their health care providers as they navigate care pathways, involving interprofessional health care teams and formal and informal caregivers, including services and resources in the community. A patient-centred clinical approach includes the physician actively inquiring into patients' circumstances and values for shared decision-making, and demonstrating empathy. It contributes to a positive, collaborative, and compassionate therapeutic relationship.	Added narrative where the physician provides the diagnosis to the patient; included patient's response and follow-up questions/concerns. Considered “What is this person's vision of a good life, and what is impeding success?” This might include their symptoms/diagnosis, movement through the health system, and structural forces counteracting their agency. These personal factors and concerns may have been added to the patient narrative and discussion questions. Reviewed for language that might cast doubt, belittle, disempower, and blame patients (e.g. “deny”, “complain”) and replaced with patient-centered language. Replaced descriptions of paternalistic decision-making with shared decision-making and respect for informed consent.

In addition to this, required and recommended readings in the cases were updated to ensure timely content that reflects decolonization efforts (as described in Table 1) and changes in scientific knowledge. Furthermore, images were diversified to challenge the assumption that white skin is the norm and to build clinical skills in identifying conditions in diverse skin tones.

In the summer of 2022, the Head of Dalhousie's health sciences library was invited to join the working group, and from there the opportunity was discussed with both liaison librarians to the Faculty of Medicine. One librarian (JP) was ultimately appointed to provide support for the project and joined the group in the fall of 2022. This support was intended to take the form of recommending library resources and conducting literature searches for the case updates and involved regular attendance at working group meetings. While the library was already accustomed to providing resource consultations and literature searching services as part of its normal offerings to faculty, becoming fully embedded in a faculty working group was less common and presented an opportunity for deeper engagement with faculty initiatives. In addition to this, the librarian had experience tutoring within the medical education CBL setting, which was an asset in supporting this work.

The librarian's integration into the Case Diversification Working Group was welcoming, and the librarian attended the next working group meeting after confirming interest in the opportunity and gaining access to working group documents and meeting materials. Since the working group had been operational prior to the librarian joining, there was a settling in period where the librarian observed conversations and determined ways to be of value to the group. In time, there was more comfort in sharing commentary during discussions on various topics, including the importance of faculty exercising critical appraisal skills rather than solely relying on point of care tools which can continually promote harmful biases [18]. Also, as an embedded member of the group, the librarian was welcome to offer suggestions on case materials which she did if she thought it helpful and within her area of expertise. She also provided on the spot recommendations to reading materials as the conversations took place, including links to the electronic copies of books held in the library's collection or journal articles that could address knowledge gaps noted during discussion.

With the librarian's expertise in resources and literature searching being the main driver for joining the group, a process gradually unfolded whereby literature searches were conducted and delivered to the working group's Chair and Reviewer when needed. While the health sciences library maintained an active literature searching service throughout this period, the librarian's integration into the working group meant she was on standby for any requests specific to this initiative, and those were prioritized as part of her regular workload. She also acted as the primary contact for the working group Chair and Reviewer when electronic resources were not working, if a one-off resource recommendation was needed, or if a case needed an updated permalink to a recommended/required reading.

All requests of the librarian were made informally and on an as-needed basis using several communication strategies such as discussion during group meetings, email, or Microsoft Teams messages. Since the librarian maintained access to working group documents, she was also assigned to find literature directly from those documents using the comments and tagging features. Timelines for delivery of results were sometimes discussed, but the librarian was confident that she would be able to prioritize searching in a timely manner since the working group was part of her portfolio. For searches that were challenging or warranted review of resources used primarily in other health professions (such as Nursing), the librarian solicited assistance and second opinions from colleagues within the health sciences library.

Providing evidence to support case revisions was challenging at times, and the variety of topics was stimulating for the librarian's searching skills (see Table 2 for the sample list of search topics explored).

**Table 2:** Sample of search topics and the curriculum unit where evidence was incorporated

Topic Searched	Overarching Unit (and Component)
Access to care/delayed care for tuberculosis in Canada	Metabolism 2 (Respirology component)
Anti-oppressive practice in mental health/psychiatry	Neuroscience (Psychiatry component)
APGAR score testing in infants with diverse skin tones (including image banks)	Metabolism 2 (Cardiology component)
Cyanosis in infants with diverse skin tones (including image banks)	Metabolism 2 (Cardiology component)
EDIA & anti-racism in nephrology and cardiology	Metabolism 2 (Cardiology and Nephrology components)
EDIA in neurology	Neuroscience (Neurology component)
Gender & racial biases in medical diagnosis/treatment/decision-making	Incorporated across all units and components
Gender inclusive language in urology	Human Development (Urology component)
Guillan-Barré syndrome and patient experience/equity	Neuroscience (Neurology component)
Impact of disclosing Parkinson's disease on patients	Neuroscience (Neurology component)
Inclusive language in male breast cancer	Human Development (Genetics component)
Multiple sclerosis (MS) and race	Neuroscience (Neurology component)
Perinatal depression in partners/fathers	Neuroscience (Psychiatry component)
Postpartum depression in queer parents	Neuroscience (Psychiatry component)

Note: Search topics were often framed as background questions, which gave the librarian flexibility in searching and provided case authors with a variety of interesting resources.

Most of the searches relied on common biomedical databases such as PubMed and Embase, but some searches required the librarian to search for grey literature or browse multimedia tools to find video content that could bolster the learning material. For example, the librarian was asked to find information on making the APGAR score more inclusive, as recognizing the visual signs of oxygen deprivation in infants with varying skin tones is extremely important for medical learners. After much searching, the librarian provided evidence discussing the implications of incorrectly scoring Black neonates [25], along with a clinical skills video from a major point of care tool showing the APGAR being performed on a white doll, but with the narrator discussing the signs of oxygen deprivation to look for in diverse skin tones.

While identifying new resources for integration into cases was of paramount importance for the librarian's role, another common task involved assisting the Reviewer with updating old links to reading materials. In some instances, this was difficult as the original cases hadn't properly cited the resource, but instead included direction to go to a resource and click a specific heading, topic, etc. This is problematic for updating materials when the library regularly evaluates its collection and makes adjustments to fit the budget and evolving needs of its faculties. As such, the original case included a link to a resource the library had not subscribed to in many years. While the librarian leveraged her connections with hospital library colleagues who had access to this resource (in hopes of identifying the exact reading material being referred to), in the end this reading (and others like it) had to be changed to work with the library's current collection.

In the spring of 2024, the Case Diversification Working Group completed updates of all cases in the first- and second-year medical curriculum across a two-year period. The revised curriculum materials have now been deployed to all pre-clerkship learners, and student feedback is regularly collected as part of curriculum delivery. Additionally, the experiences of both faculty and students who engage with these materials are being explored through qualitative research methods, and there are plans underway to continue this work through formalized processes that will include the library's involvement. Thus, the relationship between the Faculty of Medicine and the health sciences library can continue for future EDIA initiatives in the medical school, due to the positive outcome of this collaboration.

## DISCUSSION

The Case Diversification Working Group within Dalhousie University's Faculty of Medicine embarked on a mission to increase the inclusivity and patient-centredness of CBL materials within its pre-clerkship medical curricula. As part of this, a health sciences librarian was invited to join the group, and while the initial purpose of this invitation was to increase access to timely literature and resource recommendations, several opportunities for reflection emerged from this experience.

Broadly speaking, the librarian's embeddedness in the working group (and observation of monthly meetings) gave a richer perspective on the goals of the project and the types of resources needed to update materials. While the health sciences library is accustomed to conducting literature searches for faculty members, these sometimes require a short reference interview to determine the exact nature of the request, the purpose for needing the literature, and to determine time requirements and the rigour of the search. Having been embedded in the working group and observing meetings, the librarian fully understood the details of the request and the overall goals of the project and was able to jump right into searching and suggesting resources. From the perspective of the working group Chair and Reviewer, active participation of a librarian within the working group led to faster retrieval of evidence to support case revisions, with little need for additional context. In addition to this, in-depth literature searching often requires multiple attempts that include variations in terminology. Due to the existing ties between the librarian and the working group, she felt comfortable asking the Chair and Reviewer to share alternate vocabulary for key concepts and knew that informal check-ins regarding search parameters and their progress would be welcome. In searching for evidence, the librarian was sometimes surprised at how challenging it was to find what was needed, even when using comprehensive techniques that involved keyword/subject heading searching, citation chaining, and a variety of resources. The search for inclusive materials on the APGAR score is a noteworthy example of this, and after reflecting on the experience she realized her surprise at how difficult some searches were was due to her privilege, which caused her to take Medicine's consideration of her needs as a white settler for granted. Reflecting on this privilege also led to a greater understanding that there is a time and a place to deliver search results where inclusivity and patient-centredness is the goal. While health sciences librarians have positively contributed to patient rounds by providing evidence on demand [26], this working group's mandate required deeper appraisal of the evidence to ensure it did not contain outdated language or ideas that perpetuate further harm. Therefore, while the librarian initially provided links to articles in real-time during online group meetings, it was consequently determined that was no longer appropriate and literature would be provided outside the working group's meetings to ensure in-depth appraisal prior to it being considered for integration into cases.

The filtering of evidence was conducted with heavy involvement from the subject matter experts on the working group, and rich conversations between case authors also took place once the librarian delivered preliminary search results. A prominent component of this stage was consideration of who the task of appraising evidence should be assigned to, since a goal for the working group was to regularly reflect on the process and ensure group members from underrepresented communities were not disproportionately tasked with educating others. Ultimately, the gathering and reviewing of evidence was seen as part of the collective group process, as the goal was to transform the medical learning culture rather than simply tick boxes without actual change [6]. During revisions, cases were read multiple times, and the work followed a very iterative process. Thus, the librarian was sometimes relied upon to perform follow-up searches on a given topic rather than simply deliver results and close the request, and by having the working group already accounted for in the librarian's portfolio, these follow-up requests were seamless and fulfilled quickly. In addition to this, the collective group process meant that some evidence used in cases was not provided through the librarian's search methods but was sourced by those working directly on case revisions. This was a natural by-product of the group's wide-ranging expertise and interest in this initiative and led to the inclusion of supplementary resources such as dermatological image collections that are openly available online [27]. Thus, it was demonstrated through this process that traditional, scholarly evidence can co-exist alongside more mainstream resources within medical education materials.

While community consultations were part of the case diversification process, it was not possible to discuss patients' lived experience across all illnesses; nor could we expect one or two people to speak for a community or population. Incorporating evidence provided by the librarian helped the case authors introduce patient voices in a meaningful way. For example, the Neurology component of the medical curriculum includes a case about a patient who develops Guillain-Barre syndrome. The librarian completed a literature search on patient experience of people living with this syndrome, which elicited qualitative research exploring lived experiences and quality of life. Content was added about how the patient was supported by the healthcare team in hospital, and her concerns about how to take time away from work, pay her mortgage, and continue with her own caregiving responsibilities were also articulated within the materials. Therefore, the inclusion of this evidence highlights the socioeconomic impacts of illness on patients, with the goal of helping learners understand the patient perspective.

Throughout this process, reflections also took place as part of collections development work at the library. In considering how library collections can impact faculty efforts to increase inclusivity in learning materials, questions to product representatives on how their resources support EDIA in Medicine became more common. Responses to these questions were sometimes surprising given that prominent medical textbooks have been critiqued for their over-representation of white male patients in images [28], open education resources have been created to offer images depicting diversity in healthcare [29], and there are well-known information products on the market that do demonstrate a commitment to change [30]. Since more progress is evidently needed in this area, libraries should continue asking vendors how their resource aligns with calls for EDIA in healthcare, so it remains at the forefront. In addition to this, the mass updating of learning materials highlighted the importance of electronic library resources to support evidence-based curricula. Medical students have indicated that eBooks have greater convenience [31], yet high cost and electronic availability of titles [32] still create barriers for libraries to purchase in this format. To address this, librarians can continue pressing vendors on offering different format choices for key titles, engage in conversations with faculty about choosing alternate textbooks, or promote the idea of including open education resources in medical school curricula.

From a relational standpoint, it is important to acknowledge that librarians may face challenges when integrating with working groups who are initially unsure of the services they can provide. While the working relationship described here was supported by existing ties between the library and the Faculty of Medicine, an openness to growth and shared learning (by all parties involved) allowed the librarian's involvement in this initiative to succeed. For librarians who wish to support EDIA efforts at their institutions (academic or clinical) but are struggling to advocate for their abilities, beginning with key stakeholders who already value the library's offerings can be a powerful way to promote their utility in new initiatives. Adding to this, finding creative ways to demonstrate the unique value of the library is a useful place to begin when looking to expand networks and reach. For example, when liaising with patrons who share interest/engagement in EDIA-related work, creating auto alerts, helping compile reading lists, and/or establishing project accounts within databases for easy access to evidence can demonstrate high value. Additionally, reaching out to medical education departments to ask for opportunities to teach on searching for EDIA-related topics, or promoting ethical data practices for researchers studying underserved communities are also ways to support EDIA efforts while building networks. These relationships are important, as this work deserves ongoing reflection and is never finished. From a curriculum development standpoint, it is important that subject-matter expertise as well as knowledge on how to find and appraise information remain available to curriculum developers across healthcare programs. Additionally, it is also imperative that mechanisms of ongoing review of learning materials be implemented, as they are an important component of making medical curricula more inclusive [6]. Librarians have much to offer their organizations who are pursuing this work, and their efforts to facilitate access to timely, patient-centered evidence can help shape clinicians into more inclusive role models for tomorrow's health workforce.

## Data Availability

There are no data associated with this article.
